# Role of NKG2D in Obesity-Induced Adipose Tissue Inflammation and Insulin Resistance

**DOI:** 10.1371/journal.pone.0110108

**Published:** 2014-10-15

**Authors:** Jun-Jae Chung, Mary A. Markiewicz, Bojan Polić, Andrey S. Shaw

**Affiliations:** 1 Department of Pathology and Immunology, Washington University School of Medicine, St. Louis, Missouri, United States of America; 2 Department of Histology and Embryology, University of Rijeka School of Medicine, Rijeka, Croatia; 3 Howard Hughes Medical Institute, Washington University School of Medicine, St. Louis, Missouri, United States of America; National Institute of Nutrition, India

## Abstract

The early events that initiate inflammation in the adipose tissue during obesity are not well defined. It is unclear whether the recruitment of CD8 T cells to the adipose tissue during onset of obesity occurs through antigen-dependent or -independent processes. We have previously shown that interaction between NKG2D (natural-killer group 2, member D) and its ligand Rae-1ε is sufficient to recruit cytotoxic T lymphocytes to the pancreas and induce insulitis. Here, we tested whether NKG2D–NKG2D ligand interaction is also involved in obesity-induced adipose tissue inflammation and insulin resistance. We observed a significant induction of NKG2D ligand expression in the adipose tissue of obese mice, especially during the early stages of obesity. However, mice lacking NKG2D developed similar levels of insulin resistance and adipose tissue inflammation compared to control mice when placed on a high-fat diet. Moreover, overexpression of Rae-1ε in the adipose tissue did not increase immune cell infiltration to the adipose tissue either in the setting of a normal or high-fat diet. These results indicate that, unlike in the pancreas, NKG2D–NKG2D ligand interaction does not play a critical role in obesity-induced inflammation in the adipose tissue.

## Introduction

Recent studies have pointed to chronic inflammation in insulin target tissues, such as muscle, liver, and adipose tissue, as one of the causal links between obesity and insulin resistance [1]–[3]. Multiple inflammatory cytokines (TNF-α, IL-6, etc.) and signaling pathways (JNK, NF-κB) have been implicated in obesity-induced insulin resistance [4]–[10].

The adipose tissue plays a key role in regulating systemic metabolism. In addition to being a storage depot for lipids, the adipose tissue secretes a number of paracrine and endocrine factors, known as adipocytokines, that modulate metabolism and inflammation in liver, muscle, and pancreatic islets [11]. In obese mice, the secretion profile of the adipose tissue is altered by the pro-inflammatory milieu. Secretion of pro-inflammatory adipocytokines, such as resistin, IL-6, and TNF-α is increased while anti-inflammatory and insulin-sensitizing adipocytokines like adiponectin are down-regulated. It is therefore thought that dysregulation of the adipose tissue during obesity induces insulin-resistance and inflammation in major metabolic tissues.

Despite much effort, the initiating events that cause inflammation in obese adipose tissue are yet to be clearly understood. Hypoxia and ER stress have been shown to activate inflammatory signaling pathways in obese adipose tissue, but it is unclear whether they are the primary switch that triggers inflammation [12]–[16].

Various immune cell types have been implicated in obesity-induced insulin resistance [17]. Among them, macrophages are considered to be the major mediators of adipose tissue inflammation [18], [19]. Macrophages can constitute up to ∼40% of the cell number in the adipose tissue during obesity and are responsible for the majority of *Tnfa* expression in the adipose tissue [20], [21]. Additionally, in contrast to the resident macrophages found in the adipose tissue of lean mice, the macrophages recruited to the adipose tissue during obesity are pro-inflammatory [22]. The resident macrophages display characteristics of “alternatively activated” or M2 macrophages and express anti-inflammatory cytokines such as IL-10, while the newly infiltrating macrophages are of the “classically activated” or M1 category and express high levels of TNF-α and iNOS [22], [23].

More recently, T cells have emerged as a key component in obesity-induced adipose tissue inflammation. Multiple studies have reported increased numbers of CD8 T cells in the adipose tissue of obese rodents and humans along with elevated levels of IFN-γ and RANTES, which are important for T cell function and recruitment [24]–[29]. Interestingly, accumulation of T cells occurs prior to macrophage infiltration and onset of insulin resistance, and depletion of CD8 T cells alleviates adipose tissue inflammation and insulin resistance in obese mice [26], [28], [30]–[32]. This suggests that recruitment of pro-inflammatory T cells could be a primary event that initiates adipose tissue inflammation.

Still, the question of what initiates the infiltration and activation of CD8 T cells in the adipose tissue remains to be answered. It has been suggested that T cells are responding to chemokines and cytokines produced in the adipose tissue in response to cell death or hypoxia [25], [33], [34]. A more provocative idea is that certain antigens produced in the adipose tissue under obese conditions could direct immune cell responses. This hypothesis is supported by the observation that adipose tissue T cells express a restricted repertoire of T cell receptors (TCRs), whose profile is unique from that of T cells in the spleen or lymph nodes [31]–[33].

An alternative way of T cell recruitment and accumulation in tissues is through the interaction between certain high affinity receptors and their ligands independent of antigen. Our lab has previously shown that NKG2D (natural-killer group 2, member D), a cell surface receptor expressed in cytotoxic T lymphocytes (CTLs), is sufficient to induce recruitment of CTLs to pancreatic islets over-expressing its ligand and cause insulitis [35]. NKG2D binds to a number of distinct ligands which are not expressed in normal adult tissues, but up-regulated in response to stress conditions, such as oxidative stress and viral infection [36]. During obesity, the adipose tissue is subjected to various types of stresses, such as nutrient stress (from elevated glucose and lipid levels), ER stress, oxidative stress, hypoxia, and chronic inflammation [37], [38]. In this study, we examined whether NKG2D ligand expression is induced in the adipose tissue during obesity. We also tested whether interaction between NKG2D and its cognate ligands plays a role in obesity-induced adipose tissue inflammation and insulin resistance.

## Materials and Methods

### Cell Culture

3T3-L1 fibroblasts (from American Tissue Type Culture Collection) were maintained and differentiated as previously described [39]. For NKG2D ligand expression experiments, differentiated 3T3-L1 cells (day 7, ∼95% differentiated) were treated with 5 µg/ml insulin (Sigma-Aldrich, St. Louis, MO), 50 ng/ml TNF-α (Peprotech, Rocky Hill, NJ), 3 µg/ml tunicamycin (EMD Millipore, Darmstadt, Germany), 50 mU/ml glucose oxidase (Sigma-Aldrich), 100 ng/ml LPS (Invivogen, San Diego, CA), and 500 µM palmitate (Nu-Chek Prep, Elysian, MN) conjugated to 250 µM fatty acid-free BSA (SeraCare Life Sciences, Milford, MA) for 24 h.

### Mice

All mice were housed in a specific pathogen-free facility at Washington University School of Medicine. C57BL/6J and *ob*/*ob* (B6.V-*Lep^ob^*/J) mice were purchased from The Jackson Laboratory (Bar Harbor, ME). NKG2D-deficient (*Klrk1* ^-/-^) mice have been previously described [40]. The PCCALL-Rae1ε mice have been previously described [35]. Adiponectin-Cre transgenic mice were provided by Dr. P. Scherer (University of Texas Southwestern Medical Center) [41]. High-fat diet (HFD) mice were fed the D12492 60 kcal% fat diet (20 kcal% protein, 20 kcal% carbohydrate. Each gram contains 5.24 kcal, including 228 mg cholesterol from lard) from Research Diets, Inc. (New Brunswick, NJ) for up to 18 weeks. Normal chow control mice were fed a diet of 13 kcal% fat (25 kcal% protein, 62 kcal% carbohydrate. Each gram contains 3.07 kcal) from LabDiet (St. Louis, MO). Care and use of mice were conducted in accordance with protocols approved by the Animal Studies Committee at Washington University in St. Louis (Protocol Number: 20120013), in compliance with the Animal Welfare Act.

### Real-time Quantitative PCR Analysis

RNA was extracted from 3T3-L1 cells, epidydymal fat tissue, or fractionated adipocytes and stromal vascular cells with TRIzol (Life Technologies, Grand Island, NY). RNA was reverse-transcribed using random primers with the High Capacity cDNA Reverse Transcription Kit (Life Technologies). Real-time quantitative PCR was performed using the SYBR Green method on the 7500 Fast Real-Time PCR system (Life Technologies). Transcript levels were normalized to HPRT or β-actin.

The sequence of primers were: Rae-1 pan F - CCA CCT GGG AAT TCA ACA TC; Rae-1 pan R - TGA TCT TGG CTT TTC CTT GG; Mult-1 F - CAA AGG TCT GCT GCT TCA CA; Mult-1 R - TGC TTG TGT CAA CAC GGA AT; H60b F - TGC CTC AAC AAA TCG TCA TC; H60b R - CAC TCA GAC CCT GGT TGT CA; CD68 F - TAC CCA ATT CAG GGT GGA AG; CD68 R - ATG GGT ACC GTC ACA ACC TC; TNF-α F - ACG GCA TGG ATC TCA AAG AC, TNF-α R - AGA TAG CAA ATC GGC TGA CG; MCP-1 F - TCC CAA TGA GTA GGC TGG AG; MCP-1 R - TCT GGA CCC ATT CCT TCT TG; adiponectin F - GAC AAG GCC GTT CTC TTC AC; adiponectin R - CAG ACT TGG TCT CCC ACC TC; adipsin F - AGC GAT GGT ATG ATG TGC AG; adipsin R - ATT GCA AGG GTA GGG GTC TC; β-actin F - GAA GAG CTA TGA GCT GCC TGA; β-actin R - GCA CTG TGT TGG CAT AGA GGT, HPRT F - ATC AGT CAA CGG GGG ACA TA; HPRT R - AGA GGT CCT TTT CAC CAG CA.

### Fat Tissue Fractionation

The epidydymal fat tissue was isolated from euthanized mice, rinsed in PBS, weighed, and minced into small pieces (∼1 mm). The minced tissue was digested in KRH buffer (4.8 mM KCl, 2.5 mM CaCl_2_, 1.2 mM MgSO_4_, 118 mM NaCl, 20 mM HEPES [pH 7.5]) supplemented with 1.5% bovine serum albumin (BSA) (SeraCare Life Sciences), 30 µg/ml Liberase (Roche Applied Science, Indianapolis, IN), and 1 µg/ml DNaseI (Sigma-Aldrich) at 37°C for 25∼40 minutes with vigorous shaking. The digested samples were strained through a sterile 250 µm nylon mesh (Sefar, Buffalo, NY) and centrifuged at 200 g for 10 min. The floating cells were collected as the adipocyte fraction and the pelleted cells were collected as the stromal vascular fraction (SVF). The adipocytes were washed twice with KRH buffer +1.5% BSA before being processed for RNA extraction. The SVF was resuspended in ACK lysis buffer (150 mM NH_4_Cl, 10 mM KHCO_3_, 0.1 mM EDTA) and incubated at room temperature for 2 minutes to deplete erythrocytes, washed twice with KRH buffer +1.5% BSA, and finally resuspended in FACS buffer (1% BSA, 2 mM EDTA in 1×PBS) for flow cytometry or processed for RNA extraction.

### Isolation of Splenocytes and Peripheral Blood Mononuclear Cells (PBMCs)

Spleen harvested from euthanized mice were homogenized and filtered through a 40-micron cell strainer. The cells were then washed with PBS, depleted of erythrocytes with ACK lysis buffer, washed twice with PBS, and resuspended in FACS buffer.

For PBMCs, whole blood was collected from mice in EDTA-coated vacutainers (BD Biosciences, San Jose, CA). The blood was mixed with PBS and centrifuged at 300 g for 10 min. The cells were then resuspended in ACK lysis buffer, washed twice with PBS, and resuspended in FACS buffer.

### Immunofluorescent Staining and Flow Cytometry

Single-cell suspensions from the SVF of fat tissue, spleen, and blood samples were incubated in FcBlock (BD Biosciences) for 15 min at 4°C. The cells were then stained with fluorophore-conjugated antibodies for 20 min at 4°C in the dark. The antibodies were purchased from BD Biosciences (CD8-FITC, CD11c-FITC, NK1.1-FITC, CD4-PE/Cy7, CD45.2-APC), eBioscience (San Diego, CA) (NKG2D-PE, F4/80-PE/Cy7, CD11b-APC/eFluor780), or BioLegend (San Diego, CA) (CD3ε-Pacific Blue). Dead cells were excluded with 7-AAD staining (BD Biosciences). Flow cytometry data was acquired with FACSCantoII or LSRII flow cytometer (BD Biosciences) and analyzed with FlowJo (Treestar, Ashland, OR).

### Metabolic Studies

Male mice were placed on a HFD beginning at 8 weeks of age, and body weight was monitored weekly. Mice were fasted overnight (12 h) prior to measuring fasting blood glucose or performing glucose tolerance tests (GTT) and insulin tolerance tests (ITT). For GTT, glucose (1 g/kg in saline) was administered by intraperitoneal (i.p.) injection. Blood glucose levels were measured before and 15, 30, 60, 120 min after glucose injection with a handheld Contour^TS^ glucometer (Bayer, Tarrytown, NY). For ITT, insulin (0.75 U/kg, Humulin R, Lilly, Indianapolis, IN) was administered by i.p. injection and blood glucose levels were measured before and 15, 30, 60, 120 min after insulin injection.

### CTL Adoptive Transfer

CTL adoptive transfer experiments were performed as described previously [35]. Briefly, CTLs were generated *in vitro* by culturing splenocytes and lymph node cells from OT-1 T cell receptor transgenic mice in IMDM + 10% FCS with 1 µM OVA peptide (SIINFEKL) (provided by Dr. P. Allen, Washington University School of Medicine) for 5 days. Live cells were harvested using Ficoll-hypaque (GE Healthcare, Piscataway, NJ) and labeled with 1 µM CFSE (Invitrogen) and injected intravenously (10^7^ cells/mouse). Epididymal adipose tissue and spleen were harvested 24 h after injection and processed for flow cytometry.

### Statistical analyses

Data were routinely presented as mean +/- standard deviation (SD), and we determined significance by the Student's t test or ANOVA. We considered a P value of <0.05 as statistically significant.

## Results

### Induction of NKG2D ligands in fat tissue of obese mice

**Figure 1:**
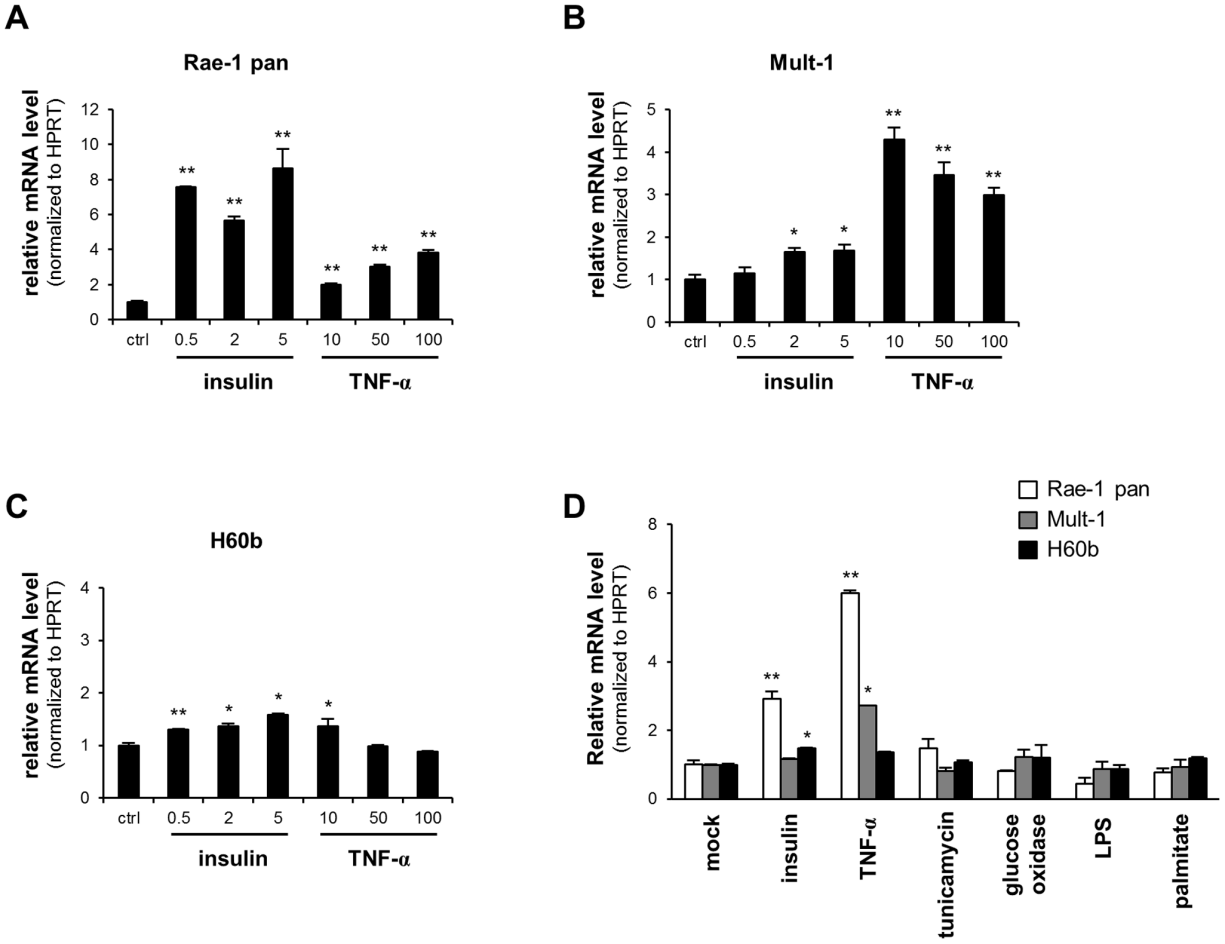
Insulin and TNF-α increases NKG2D ligand expression in 3T3-L1 adipocytes. (A-C) Differentiated 3T3-L1 cells were treated with insulin (0.5–5 µg/ml) or TNF-α (10–100 ng/ml) for 24 hours. The cells were then analyzed by quantitative PCR to determine the expression levels of (A) Rae-1, (B) Mult-1, and (C) H60b. (D) Differentiated 3T3-L1 cells were treated with insulin, TNF-α, tunicamycin, glucose oxidase, LPS, and palmitate. The cells were then analyzed by quantitative PCR for NKG2D ligand expression. The results were normalized to HPRT levels and are shown as fold-increase over control. The data represent mean ± SD of 3 independent experiments. * p<0.05, ** p<0.01 vs. control.

NKG2D binds to a number of distinct ligands, which are usually not expressed in normal adult tissues, but are up-regulated in response to various ‘cellular stress conditions’, such as oxidative stress, viral infection, and DNA damage [42]. The induced ligands are thought to act as a signal to the immune system for the removal of damaged cells. In order to identify factors that could potentially regulate NKG2D ligands in adipocytes, we incubated differentiated 3T3-L1 cells with factors that induce various cell responses. Insulin and TNF-α treatment caused a significant increase in the mRNA levels of different NKG2D ligands at varying concentrations. Insulin had the greatest effect on Rae-1 mRNA level among the NKG2D ligands (Fig. 1A) while TNF-α treatment increased both Rae-1 and Mult-1 (Fig. 1A and B). H60b mRNA levels were only moderately affected by insulin and TNF-α (Fig. 1C). Meanwhile, incubation in tunicamycin, glucose oxidase, LPS, and palmitate did not have an effect (Fig. 1D).

**Figure 2:**
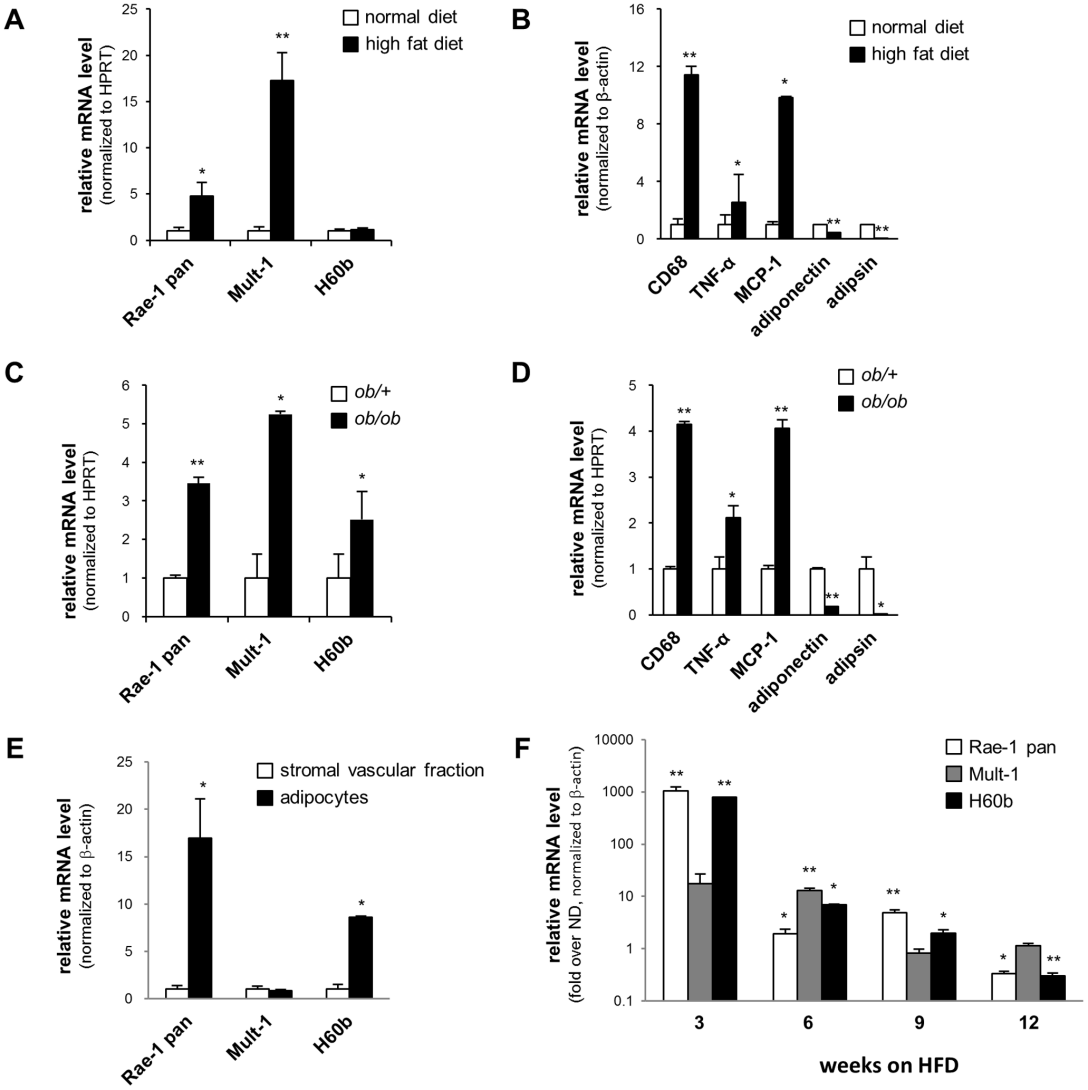
NKG2D ligands are increased in the adipose tissue of obese mice. Expression of NKG2D ligands in the epididymal adipose tissue from (A) DIO-mice (8 weeks on HFD) and (C) ob/ob mice was determined by quantitative PCR. mRNA levels of CD68, TNF-α, MCP-1, adiponectin, and adipsin were measured in (B) DIO-mice and (D) ob/ob mice for control. (E) The epididymal adipose tissue of DIO-mice (6 weeks on HFD) was fractionated into the stromal vascular fraction and adipocytes and analyzed by quantitative PCR for expression of NKG2D ligands. (F) Adipocytes were obtained from the epipdidymal adipose tissue of DIO-mice at different time points after starting high-fat diet. The adipocytes were then analyzed by quantitative PCR for expression of NKG2D ligands. The results were normalized to (A–D) HPRT or (E, F) β-actin levels and are shown as fold-increase over (A–D, F) lean control mice or (E) stromal vascular fraction. The data represent mean ± SD of 3 independent experiments. * p<0.05, ** p<0.01 vs. (A–D, F) lean control mice or (E) stromal vascular fraction.

Insulin and TNF-α levels are known to be elevated during obesity in humans and rodents. To address whether NKG2D ligands are induced by obesity, we analyzed the adipose tissue of diet-induced obese (DIO) mice. RNA was isolated from epididymal adipose tissue obtained from mice fed either a normal diet (ND) or a high-fat diet (HFD) and analyzed by real-time qPCR. Rae-1 and Mult-1 mRNA levels were increased approximately 5-fold and 17-fold respectively in the DIO-mice compared to lean control mice. (Fig. 2A). H60b expression level was not altered in DIO-mice. As control, we confirmed increased expression of CD68, TNF-α, and MCP-1 and decreased expression of adiponectin and adipsin in DIO-mice (Fig. 2B).

To confirm these results, we also analyzed the adipose tissue of *ob/ob* mice, a genetic model of obesity (Fig. 2C, D). Similar to the DIO-mice, Rae-1 and Mult-1 transcript levels were increased in the *ob/ob* mice compared to lean *ob/+* controls (Fig. 2C). In addition, H60b mRNA level was also slightly increased in the ob/ob mice (Fig. 2C).

The adipose tissue is composed of adipocytes, immune cells, vascular cells, and undifferentiated preadipocytes. In order to determine which cell types express NKG2D ligands, the epidydymal adipose tissue of DIO-mice was fractionated into adipocytes and the stromal vascular fraction (SVF) via collagenase digestion. Rae-1 was expressed predominantly in the adipocyte fraction, while Mult-1 had similar expression levels in the adipocytes and the SVF (Fig. 2E). H60b expression was higher in the adipocyte fraction (Fig. 2E).

Induction of NKG2D ligands in the adipose tissue of obese mice led us to hypothesize that this could be a mechanism by which CD8 T cells are recruited to the adipose tissue to initiate inflammation during obesity. Infiltration of CD8 T cells to the adipose tissue of DIO-mice has been shown to occur relatively soon after starting the HFD regimen (4∼5 weeks after initiation of diet). In order to see whether NKG2D ligand induction occurs within a similar time frame, we measured the time-course kinetics of NKG2D ligand expression in DIO-mice (Fig. 2F). The mRNA levels of Rae-1 and Mult-1 were highest at the earliest time-point measured (3 weeks post-initiation of diet) and gradually decreased during the progression of obesity. These results suggest that early changes that occur in the adipose tissue of mice on a HFD induce the expression of NKG2D ligands.

#### Obesity-induced insulin resistance and adipose tissue inflammation in NKG2D-deficient mice

The NKG2D–deficient (*klrk^-/-^*) mice lack the expression of NKG2D in all immune cells including CD8 T cells. To directly test whether NKG2D ligands are involved in the development of obesity-induced insulin resistance, we placed wild-type and *klrk^-/-^* mice on a HFD. Age-matched male mice were divided into two groups and placed on either ND or HFD. We measured the changes in body weight and fasting glucose levels for 18 weeks. In order to measure insulin sensitivity, we performed glucose tolerance test (GTT) and insulin tolerance test (ITT) at 6, 12, and 18 weeks after starting the diet regimen.

**Figure 3:**
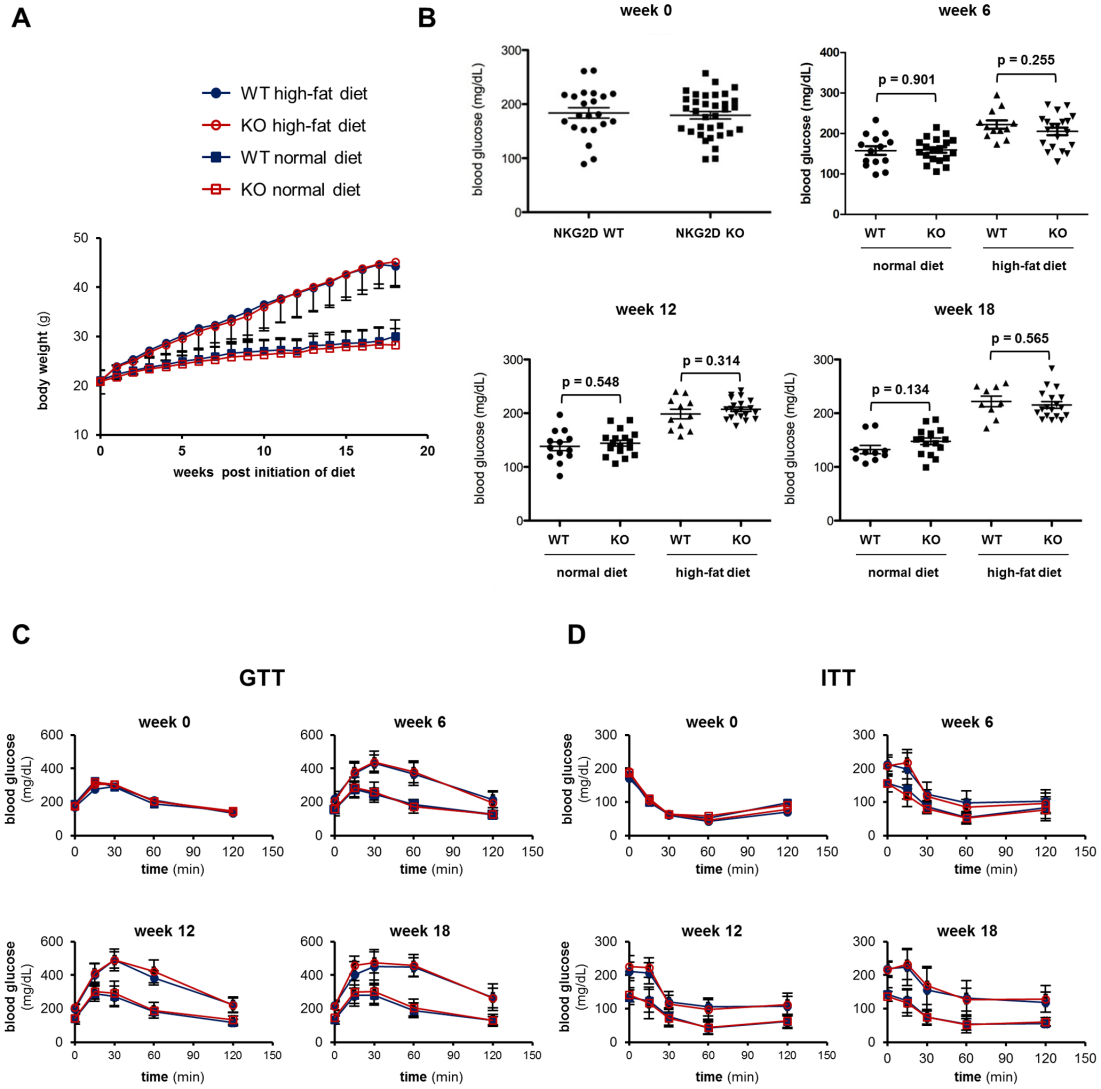
Susceptibility to obesity-induced insulin resistance is not altered in klrk^-/-^ mice. (A) Body weight of WT and klrk^-/-^ mice placed on normal diet or high-fat diet for 18 weeks. (B) Fasting blood glucose levels measured after 0, 6, 12, and 18 weeks on high-fat diet. (C) Glucose tolerance test and (D) insulin tolerance test performed after 0, 6, 12, and 18 weeks on high-fat diet. The numbers of mice used for each experiments were at least 10 for WT normal diet, at least 9 for WT high-fat diet, at least 16 for klrk^-/-^ normal diet, and at least 18 for klrk^-/-^ high-fat diet.

At baseline (8-weeks old), there was no difference between WT and *klrk^-/-^* mice in body weight (Fig. 3A) and fasting blood glucose levels (Fig. 3B). Rapidly within a few weeks, mice on the HFD began to outweigh mice on the normal diet, and the gap between the two groups continued to grow until the end of the experiment. There was no difference in body weight change between WT and *klrk^-/-^* mice in either the ND or HFD group throughout the experiment.

In GTT experiments (Fig. 3C), obese mice were significantly less tolerant compared to lean mice in both WT and *klrk^-/-^* mice. However, there was no difference between WT and *klrk^-/-^* mice in any of the time points. Similar results were obtained in ITT assays (Fig. 3D) with WT and *klrk^-/-^* mice displaying similar degrees of insulin sensitivity. Fasting glucose, which is also indicative of insulin sensitivity, was not altered in *klrk^-/-^* mice. (Fig. 3B)

**Figure 4:**
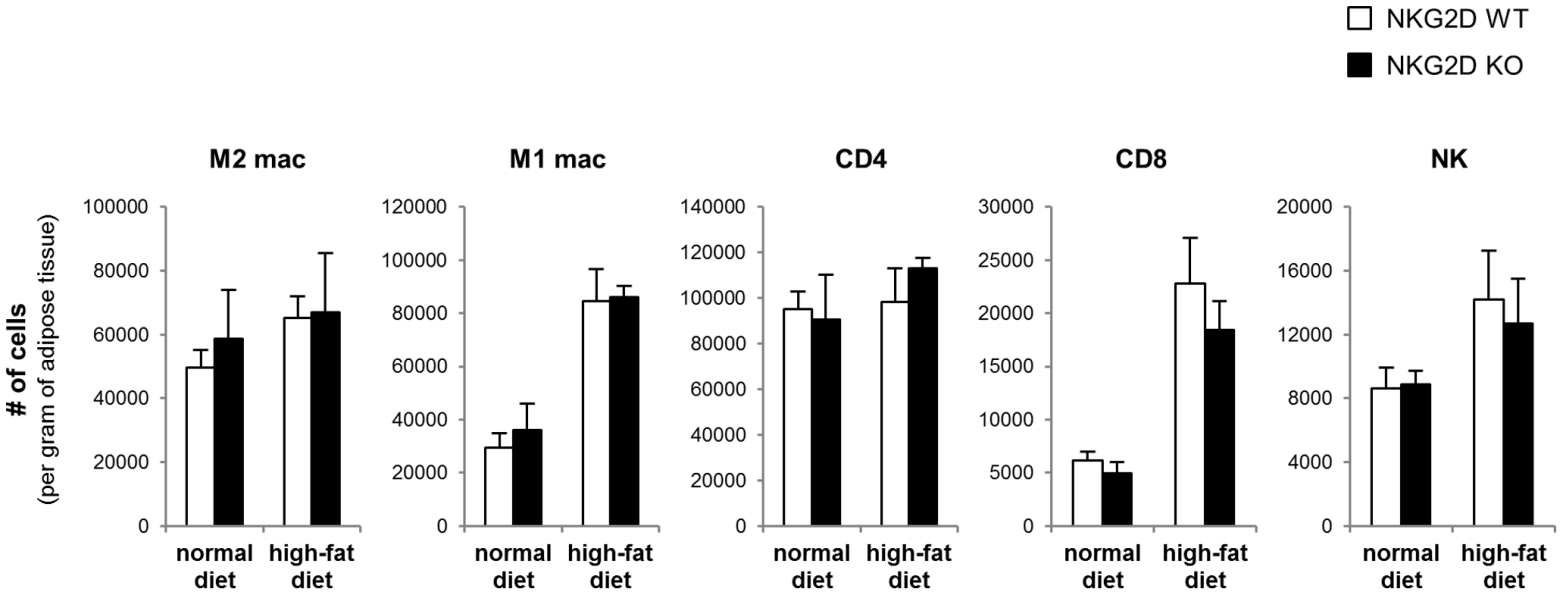
Immune cell infiltration of adipose tissue during obesity is not altered in klrk^-/-^ mice. The stromal vascular fraction was isolated from the epididymal adipose tissue of DIO and lean mice after 18 weeks on the diet regimen. The cells were stained with antibodies to identify M1 macrophages (F4/80^+^, CD11c^+^), M2 macrophages (F4/80^+^, CD11c^-^), CD4 T cells (CD3ε^+^, CD4^+^), CD8 T cells (CD3ε^+^, CD8^+^), and NK cells (CD3ε^-^, NK1.1^+^) and analyzed by flow cytometry. The cells were gated on 7AAD^−^, CD45^+^ populations. The data represent mean ± SD of 3 independent experiments.

At the end of 18 weeks on the diet regimen, the epididymal adipose tissue was harvested to analyze immune cell infiltration. The SVF was isolated by collagenase digestion, stained for cell surface markers, and analyzed by flow cytometry. Consistent with previous reports, there was a significant increase in the number of F4/80^+^CD11c^+^ M1 macrophages and CD8 T cells in the adipose tissue of HFD-fed mice compared to lean counterparts (Fig. 4). However, the number of M1 macrophages and CD8 T cells in the adipose tissue were similar in WT and *klrk^-/-^* mice. We also confirmed that there was no difference in CD8 T cell infiltration after 3 weeks of HFD, when NKG2D ligand expression was highest (data not shown). These results indicate that NKG2D does not affect CD8 T cell recruitment to the adipose tissue during obesity.

#### Obesity-induced adipose tissue inflammation in fat-specific Rae-1ε transgenic mice

**Figure 5:**
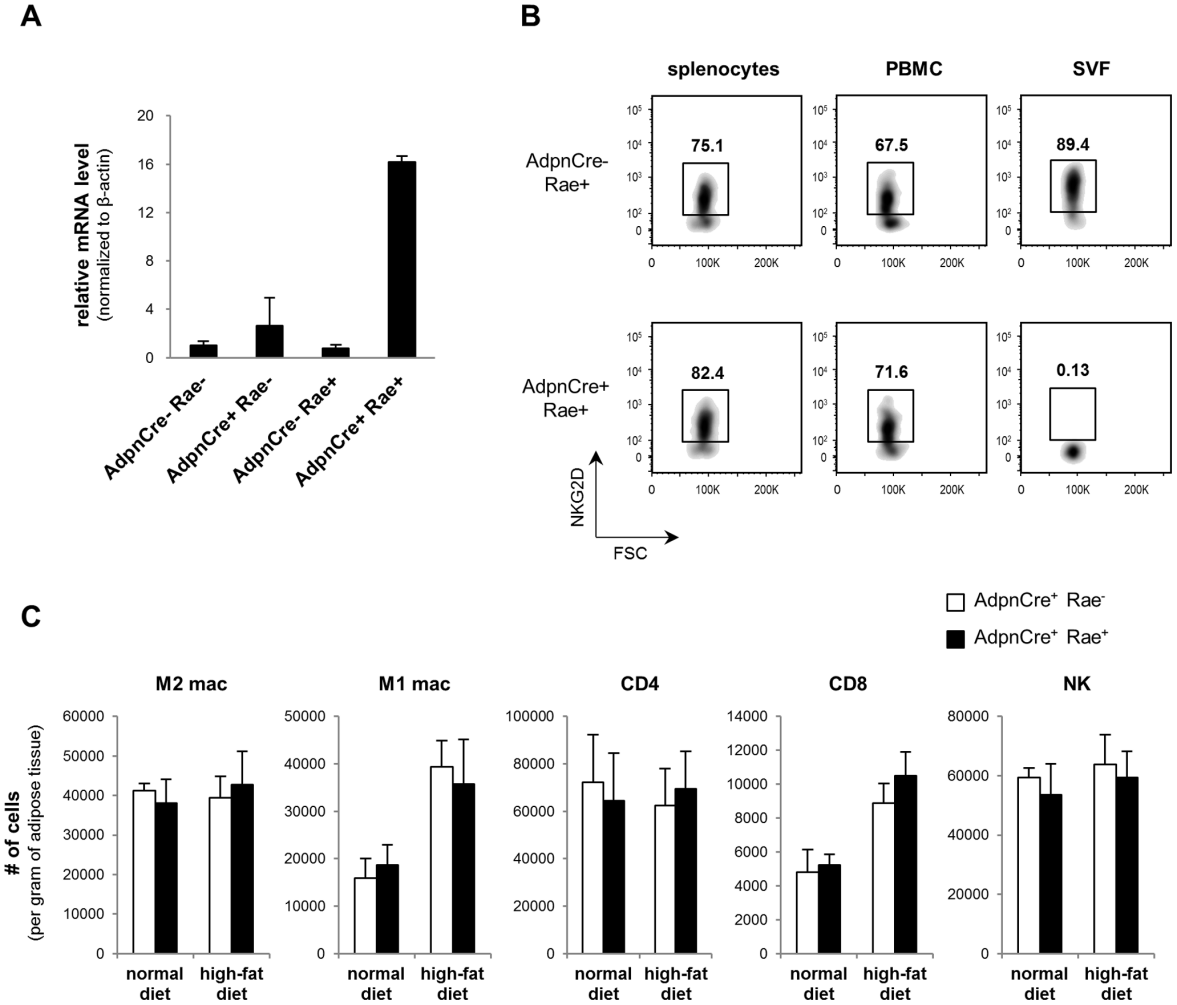
Immune cell infiltration of adipose tissue during obesity is not altered in AdpnCre^+^ Rae^+^ mice. (A) The epididymal adipose tissue from AdpnCre^+^ Rae^+^ and control mice were analyzed by quantitative PCR to determine Rae-1 expression. The results were normalized to β-actin levels and are shown as fold-increase over AdpnCre^−^ Rae^−^ mice. (B) Splenocytes, peripheral blood mononuclear cells (PBMC), and stromal vascular fractions isolated from the epididymal adipose tissue of AdpnCre^−^ Rae^+^ and AdpnCre^+^ Rae^+^ mice were stained with NKG2D antibodies and analyzed by flow cytometry. Cells were gated on 7AAD^−^, CD45^+^, CD3ε^−^, NK1.1^+^ populations. (C) The stromal vascular fraction isolated from the epididymal adipose tissue of AdpnCre^+^ Rae^−^ and AdpnCre^+^ Rae^+^ mice after 5 weeks on the diet regimen were stained with antibodies and analyzed by flow cytometry. The cells were gated on 7AAD^−^, CD45^+^ populations. The data represent mean ± SD of 3 independent experiments.

In order to confirm these results, we generated a transgenic mice specifically over-expressing Rae-1ε in the adipose tissue (AdpnCre^+^ Rae^+^) by crossing adiponectin-Cre mice [41] with PCCALL-Rae1ε mice [35]. Real-time qPCR was used to confirm Rae-1ε over-expression in the adipose tissue (Fig. 5A). Rae-1 mRNA levels were not altered in liver and muscle (data not shown), indicating specific overexpression of Rae-1ε in the adipose tissue. It is known that NKG2D expression in NK cells and T cells is down-regulated in transgenic mice constitutively overexpressing NKG2D ligands [43], [44]. Indeed, we observed loss of NKG2D expression in NK cells isolated from the adipose tissue, but not spleen or blood, again confirming fat-specific overexpression of Rae-1 in the Adpn^+^ Rae^+^ mice (Fig. 5B).

To examine the early events of obesity-induced immune cell infiltration, these transgenic mice (AdpnCre^+^ Rae^+^) were placed on a high-fat diet for 5 weeks, along with age-matched control mice lacking the transgene (AdpnCre^+^ Rae^−^). At the end of 5 weeks, the SVF from the adipose tissue were analyzed by flow cytometry. Adipose tissue from obese mice contained approximately twice the number of M1 macrophages and CD8 T cells compared to lean mice (Fig. 5C). However, the number of CD8 T cells and M1 macrophages in the adipose tissue was not altered by fat-specific overexpression of Rae-1ε. These results are consistent with the observations made in *klrk^-/-^* mice and confirm that NKG2D is not involved in the recruitment of CD8 T cells to the adipose tissue during obesity.

**Figure 6:**
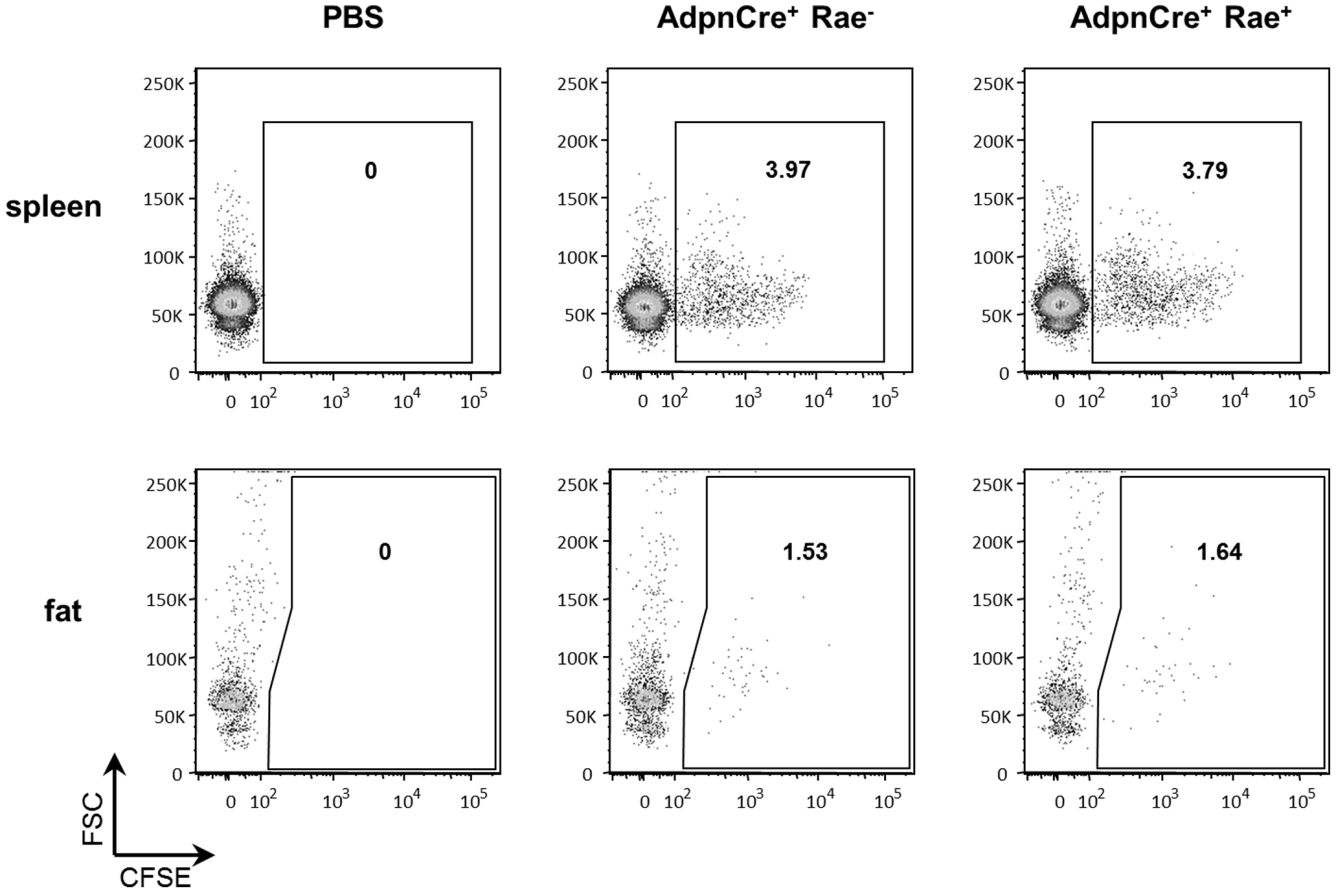
Rae-1ε overexpression does not induce CTL accumulation in the adipose tissue. CTLs generated from OT-1 mice were labeled with CFSE and injected intravenously. A group of mice were injected with PBS for control. Spleen and epididymal adipose tissue were isolated 24 hours after injection and analyzed by flow cytometry. Cells were gated on 7AAD^−^, CD45^+^, CD3ε^+^ populations. The data are representative of at least 3 individual mice per group.

The *klrk^-/-^* mice and AdpnCre^+^ Rae^+^ mice used in the previous experiments were housed in specific pathogen-free conditions, and therefore, the number of circulating CTLs is minimal. In mice, NKG2D is expressed in activated CTLs, but not in naïve CD8 T cells. We therefore speculated that the lack of a phenotype could be due to low numbers of circulating CTLs. In addition, it was possible that the constitutive overexpression of Rae-1ε was down-regulating NKG2D expression in circulating T cells. To circumvent these issues, and to directly test whether up-regulation of NKG2D ligands can induce recruitment and accumulation of cytotoxic CD8 T cells in the adipose tissue, we intravenously injected CFSE-labeled CTLs into control and AdpnCre^+^ Rae^+^ mice. 24 hours after injection, we measured the number of CFSE^+^ CTLs recruited to the spleen and adipose tissue (Fig. 6). Similar numbers of CFSE^+^ CTLs were detected in the spleen of AdpnCre^+^ Rae^−^ and AdpnCre^+^ Rae^+^ mice, indicating that equal number of CTLs was successfully transferred to each mouse. In the adipose tissue, the overexpression of Rae-1ε did not result in increased recruitment of CTLs. These results demonstrate that, in contrast to pancreatic islets, NKG2D-NKG2D ligand interaction is not sufficient to drive accumulation of NKG2D-expressing CTLs in the adipose tissue.

## Discussion

In this study, we tested a model of antigen-independent recruitment of CD8^+^ T cells to the adipose tissue during obesity via NKG2D-NKG2D ligand engagement. Results from our experiments suggest that interaction between the immunoreceptor NKG2D and its cognate ligands is not critically involved in the development of adipose tissue inflammation during diet-induced obesity. Neither deletion of NKG2D nor fat-specific overexpression of an NKG2D ligand had significant effects on insulin sensitivity or infiltration of immune cells to the adipose tissue in the setting of a high-fat diet.

The non-development of phenotype does not seem to be due to strain-specificity of the mice used in our experiments. The composition of immune cells is known to vary among different strains of mice [45]. In addition, the immune system is compromised in certain genetic obese mice models, such as the *ob/ob* mice or the *db/db* mice, because leptin is required for immune cell homeostasis, especially T cells [46]. In order to avoid such variables, we chose to utilize the diet-induced obese (DIO)-mouse model using C57BL/6 mice. The C57BL/6 strain is the predominant strain used to study nutrient-dependent metabolic disorders in the literature and was the strain used to demonstrate the involvement of various immune cells in obesity-induced inflammation.

The NKG2D-deficient mice display a NK cell hyperproliferation phenotype [40], which could be a confounding factor in the case that NK cells are also involved in adipose tissue inflammation. However, as of date, NK cells have not been clearly implicated in obesity-induced insulin resistance. There have been conflicting reports of changes in the number of NK cells in the adipose tissue during obesity. In our experiments, we did not observe a clear reproducible pattern of NK cell recruitment.

Similar to the NKG2D-deficient mice, we did not observe any difference in immune cell infiltration in the fat-specific Rae-1ε mice. We could not directly determine the protein level of Rae-1ε in the adipocytes of the fat-specific Rae-1ε mice due to the lack of antibodies suitable for immunohistochemistry or Western blots and the technical difficulties in performing flow cytometry with adipocytes. However, we did observe a nearly complete down-regulation of NKG2D specifically in NK cells isolated from the adipose tissue, but not in cells isolated from spleen or blood. It is well known that engagement of Rae1ε by NKG2D results in NKG2D down-regulation [37], [43], [47]. Therefore, these results show that the level of transgene expression was sufficient to completely quench NKG2D expression on NK cells in the adipose tissue, suggesting that Rae-1ε is being expressed at biologically-relevant levels.

Although we observed induction of both Rae-1 and Mult-1 in obese mice, only Rae-1 was expressed predominantly in fat cells. It should be noted that adipocyte fractions isolated from adipose tissue of obese mice are often contaminated with lipid-laden immune cells, which could have affected the outcome of the NKG2D ligand expression analyses. Nonetheless, we speculated that overexpressing Rae-1ε in the adipose tissue would closely resemble the expression pattern of NKG2D ligands during obesity. It is possible that overexpressing Mult-1 instead could have yielded different results.

It has been shown that circulating levels of CD8^+^ T cells in male C57BL/6 mice are relatively low compared to other mouse strains. In addition, NKG2D is only expressed in activated CD8^+^ T cells, the number of which may not be sufficient in mice raised in a SPF facility. In order to circumvent these issues, we adoptively transferred activated CTLs into the fat-specific Rae-1ε transgenic mice. Contrary to our expectations, overexpression of Rae-1ε in adipocytes did not lead to accumulation of CTLs in the adipose tissue. This is in contrast to previous results from similar experiments performed in the pancreatic islets [35]. This may be due to differences in the architecture of the vasculature between the pancreas and the adipose tissue, which could affect how surface proteins are detected by lymphocytes in circulation.

Despite the lack of evidence for the involvement of NKG2D in obesity-induced inflammation and insulin resistance, it is still interesting that NKG2D ligands are up-regulated in the adipose tissue in response to a high fat diet. In fact, the human NKG2D ligands MICA and MICB have been shown to be induced in the liver of obese patients [48]. In addition, NKG2D ligands are induced in the aorta of *ApoE* ^-/-^ mice fed a western diet and are involved in atherosclerotic plaque formation [38]. The nature of the response of the immune system to NKG2D ligand induction necessitates a tight regulation of its expression. Previous studies have shown that various ‘cell stress’ conditions, including DNA damage and viral infection, can induce NKG2D ligand expression. However, it seems that such cues are not sufficient to up-regulate NKG2D ligands in all cell types. It is more likely that different signals trigger the expression of the multiple NKG2D ligands in different cell types. Indeed, insulin and TNF-α were able to significantly increase transcript levels of Rae-1 and Mult-1, but not H60b, in differentiated 3T3-L1 adipocytes. Notably, high-fat diet resulted in the up-regulation of the same subgroup of NKG2D ligands, namely Rae-1 and Mult-1. Therefore, it is tempting to speculate that these signals are also responsible for the induction of NKG2D ligands in the adipose tissue *in vivo* given that insulin and TNF-α levels are known to be elevated in obesity.

Interestingly, multiple NKG2D ligands were expressed in undifferentiated 3T3-L1 cells and the expression levels decreased after differentiation (data not shown). This could be attributed to the cease of proliferation that occurs when 3T3-L1 cells differentiate. It was recently demonstrated that Rae-1 expression is regulated by the transcription factor E2F, which is associated with cell cycle. On the other hand, it is also possible that NKG2D ligand expression is actively repressed by the differentiation process. Identification of such factors would shed light on how the strict regulation of NKG2D ligands is achieved in various cell types.

The biological significance of NKG2D ligand induction in the adipose tissue during obesity is unclear. Adipocytes in the adipose tissue of HFD-fed mice are subjected to various cell stress conditions due to increased metabolic loads, hypoxic conditions, and proinflammatory environment. Hence, the most obvious explanation would be that NKG2D ligands are being expressed by damaged adipocytes. It should be noted that the different types of NKG2D ligands do not seem to respond in the same manner. Only Rae-1 and Mult-1 were induced by the introduction of the high-fat diet while H60b levels remained the same. Furthermore, Rae-1 was predominantly found in adipocytes whereas Mult-1 expression levels were similar in adipocytes and stromal vascular cells. How these differences affect adipose tissue homeostasis and/or pathology during obesity remain to be determined.

In summary, our results rule out the interaction between NKG2D and its ligands as a possible mechanism of CD8^+^ T cell recruitment to the adipose tissue during obesity. Our findings regarding the induction of NKG2D ligands in the adipose tissue during obesity provide a new model to investigate the mechanisms involved in the regulation of NKG2D ligand expression.
